# Hypoxia and hypoxia-inducible factors in diabetes and its complications

**DOI:** 10.1007/s00125-021-05380-z

**Published:** 2021-01-26

**Authors:** Sergiu-Bogdan Catrina, Xiaowei Zheng

**Affiliations:** 1 Department of Molecular Medicine and Surgery, Karolinska Institutet, Stockholm, Sweden; 2 Department of Endocrinology and Diabetes, Karolinska University Hospital, Stockholm, Sweden; 3 Center for Diabetes, Academic Specialist Centrum, Stockholm, Sweden

**Keywords:** Diabetes, Diabetes complications, Diabetic foot ulcer, Diabetic nephropathy, Diabetic retinopathy, Hypoxia, Hypoxia-inducible factor, Insulin resistance, Prolyl hydroxylase inhibitor, Review

## Abstract

Hypoxia-inducible factors (HIFs) are the key regulators of oxygen homeostasis in response to hypoxia. In diabetes, multiple tissues are hypoxic but adaptive responses to hypoxia are impaired due to insufficient activation of HIF signalling, which results from inhibition of HIF-1α stability and function due to hyperglycaemia and elevated fatty acid levels. In this review, we will summarise and discuss current findings about the regulation of HIF signalling in diabetes and the pathogenic roles of hypoxia and dysregulated HIF signalling in the development of diabetes and its complications. The therapeutic potential of targeting HIF signalling for the prevention and treatment of diabetes and related complications is also discussed.

**Supplementary Information:**

The online version contains a slideset of the figures for download, available at 10.1007/s00125-021-05380-z.

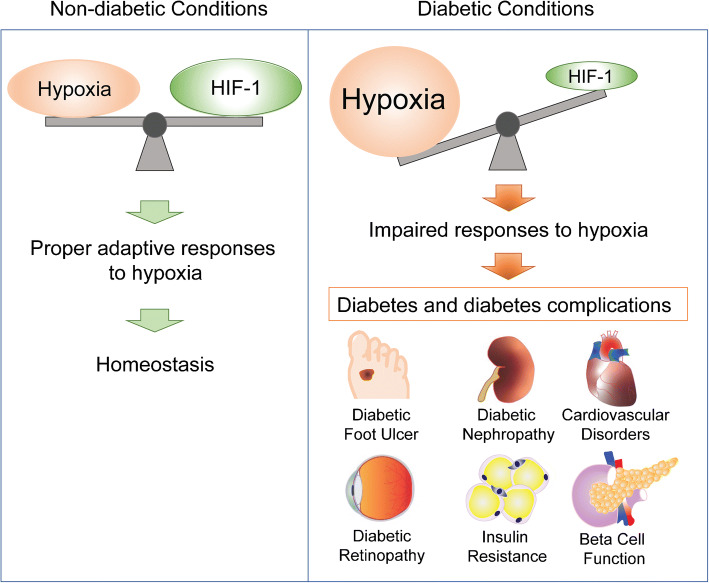

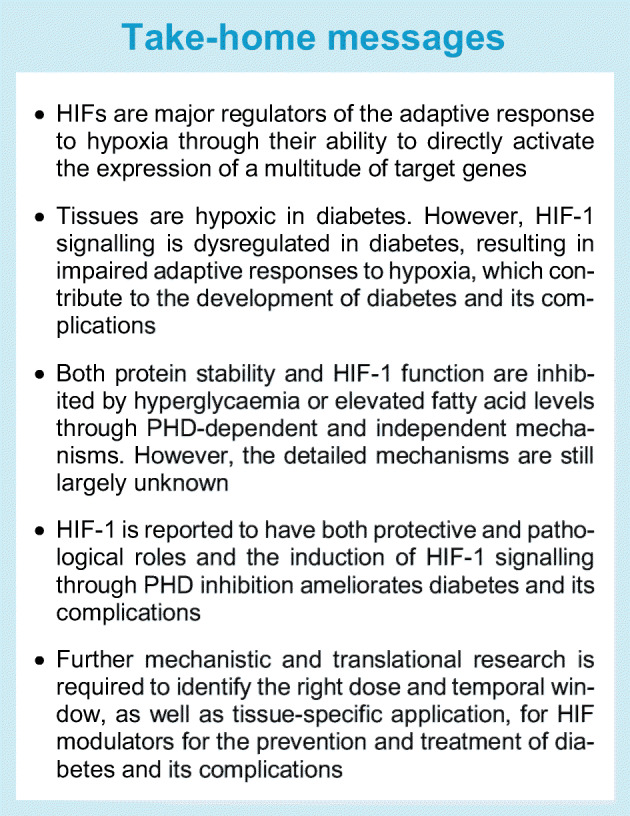



## Hypoxia and adaptive responses to hypoxia

Maintenance of oxygen homeostasis is fundamental for all cells of metazoan organisms. Hypoxia occurs when oxygen consumption exceeds oxygen supply. Complex adaptive mechanisms have developed to facilitate cell survival during hypoxia, such as metabolic reprogramming of mitochondrial oxidative phosphorylation to anaerobic glycolysis, increased erythropoiesis and angiogenesis, proliferation, differentiation and migration. Impaired adaptive responses to hypoxia contribute to the pathophysiology of many diseases [1].

## Hypoxia-inducible factors

Hypoxia-inducible factors (HIFs) are major regulators of adaptive responses to hypoxia. As transcription factors, HIFs directly activate the expression of several hundred target genes to maintain cellular oxygen homeostasis [1]. HIF signalling can also repress gene expression, mostly indirectly via HIF target genes such as transcriptional repressors and microRNAs [1]. HIF-induced adaptive responses to hypoxia are protective in many diseases but can be detrimental, such as when they promote cancer progression. HIF signalling also interacts with other signalling pathways, such as Notch and NF-κB, to regulate responses to hypoxia [2].

HIFs are heterodimeric proteins, consisting of an oxygen-sensitive α subunit and a constitutively expressed HIF-1β subunit. There are three isoforms of HIF-α, namely, HIF-1α, HIF-2α and HIF-3α. HIF-1α is ubiquitously expressed but HIF-2α and HIF-3α are tissue-specific. HIF-1 is induced early following the onset of hypoxia, whereas the activation of HIF-2 is usually slower and is sustained for longer [3]. Despite acting through a common hypoxia response element (HRE), HIF-1 and HIF-2 can activate distinct target genes depending on the cellular context [4]; HIF-3 is less well-studied.

## Regulation of HIFs

**Fig. 1:**
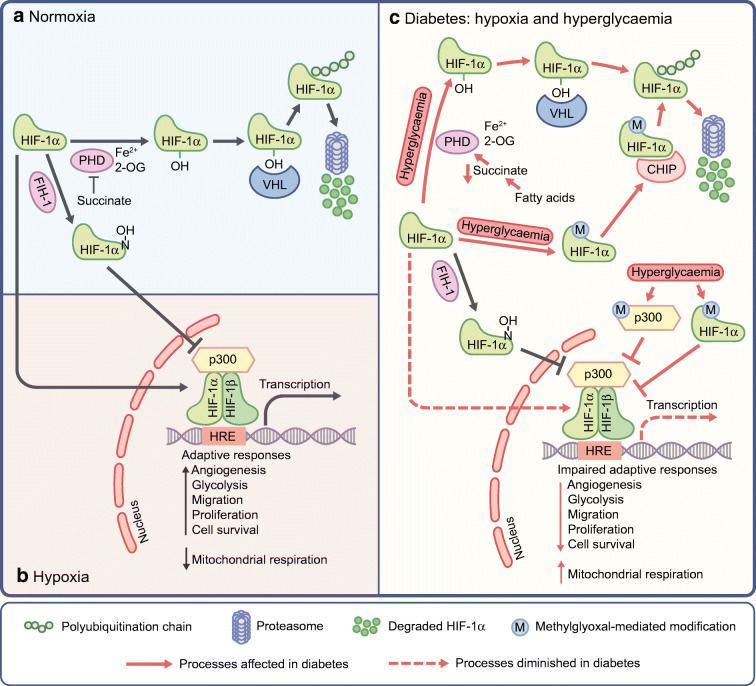
Regulation of HIF-1 under non-diabetic and diabetic conditions. (**a**) In normoxia, HIF-1α is hydroxylated by PHD in the presence of Fe^2+^ and 2-oxoglutarate (2-OG). The hydroxylated HIF-1α is recognised by VHL, which mediates the ubiquitylation and proteasomal degradation of HIF-1α. Succinate can inhibit the PHD-mediated degradation of HIF-1α. HIF-1α can also be hydroxylated at an asparagine residue (N803) by factor-inhibiting HIF-1 (FIH-1), which prevents the recruitment of coactivators. (**b**) Under hypoxic and non-diabetic conditions, HIF-1α is stabilised and translocates to the nucleus, where it dimerises with HIF-1β on the HRE of target genes, recruits coactivators including p300, and activates the transcription of HIF-1 target genes that mediate adaptive responses to hypoxia. (**c**) In diabetes, despite profound hypoxia, HIF-1α stability and function are inhibited, leading to insufficient HIF-1 activation and, therefore, impaired adaptive responses to hypoxia. Mechanistically, hyperglycaemia not only promotes PHD-mediated HIF-1α degradation but also induces methylglyoxal-mediated modification of HIF-1α, which facilitates the carboxy terminus of Hsp70-interacting protein (CHIP)-mediated HIF-1α ubiquitylation. Methylglyoxal also inhibits HIF-1 dimerisation and coactivator recruitment through modification of p300. Moreover, elevated fatty acids can promote PHD-mediated HIF-1α degradation by decreasing succinate levels in type 2 diabetes. This figure is available as part of a downloadable slideset

The regulation of HIF signalling has been recently reviewed [5]. Although the transcription and translation of HIF genes are regulated by various mechanisms, HIF signalling is largely regulated at post-translational levels. The protein stability of HIF-α is tightly regulated by oxygen and the t_½_ of HIF-1α is less than 5 min upon reoxygenation. The regulation of HIF-1α under conditions of normoxia is depicted in Fig. 1a. HIF-1α is hydroxylated in the presence of oxygen, 2-oxoglutarate and iron by prolyl hydroxylase domain proteins (PHDs) 1–4. Hydroxylation occurs on the two conserved proline residues (P402 and P564) in the oxygen-dependent degradation domain. Hydroxylated HIF-1α is ubiquitylated by the von Hippel–Lindau protein (VHL), a ubiquitin E3 ligase, before being degraded by the 26S proteasome. Upon hypoxia, HIF-α is stabilised and translocates to the nucleus, where it dimerises with HIF-1β, binds to the conserved HRE in HIF target genes, and activates gene transcription (Fig. 1b). The transactivation activity of HIF is also regulated by oxygen, mediated by factor-inhibiting HIF-1 through the hydroxylation of an asparagine residue (N803), which prevents the recruitment of coactivators CREB-binding protein (CBP) and p300. HIF-α stability can also be regulated by oxygen-independent pathways, such as heat shock protein (Hsp) 90 and receptor for activated C kinase 1, as well as p53 and glycogen synthase kinase 3β-mediated mechanisms. Other post-translational modifications, such as phosphorylation, SUMOylation, deubiquitylation and epigenetic modification, can also regulate HIF signalling (reviewed in [5]). HIF-1 signalling can also be regulated by dozens of other proteins, many of which are HIF-1 target genes and participate in feed-forward or feedback regulation of HIF-1 function [2].

## Dysregulation of HIF-1 signalling in diabetes

Emerging evidence indicates that in diabetes, tissues such as the retina [6], kidney [7, 8], pancreatic islets [9], adipose [10], skin and wounds [11] are hypoxic, suggesting that hypoxia plays a central role in the development of diabetes and diabetes complications. However, HIF-1-mediated adaptive responses to hypoxia are impaired in diabetes, leading to cellular dysfunction. The underlying mechanisms are still not completely understood.

Several studies have shown that HIF-1 activation in diabetic tissues is submaximal for the observed degree of hypoxia, due to the repression of HIF-1 signalling. Both protein stabilisation and transactivation activity of HIF-1 are inhibited in wounds [11, 12], kidney [7] and heart [13, 14] of individuals with diabetes and in animal models of diabetes. Hyperglycaemia is, by definition of the disease, common for both type 1 and type 2 diabetes. High glucose levels inhibit the stabilisation and function of HIF-1 under hypoxic conditions in dermal fibroblasts and endothelial cells [11, 12], cardiomyocytes [13], retinal epithelial cells [15] and proximal tubular cells [16]. The inhibitory effects of hyperglycaemia on HIF-1α stability is p53-independent, since high glucose can still destabilise HIF-1α during hypoxia in p53^−/−^ fibroblasts [12]. However, PHD inhibition or VHL inactivation can largely rescue HIF-1α stability [11], indicating an important role for PHD and VHL in mediating the degradation of HIF-1α during hyperglycaemia (Fig. 1c). However, the underlying mechanisms facilitating PHD- and VHL-mediated HIF-1α degradation during hyperglycaemia are still not fully understood.

Hyperglycaemia also induces the accumulation of methylglyoxal, which mediates HIF-1α destabilisation in a PHD- or VHL-independent manner (Fig. 1c). Methylglyoxal increases the interaction of HIF-1α with Hsp40 and Hsp70, facilitating the ubiquitylation of HIF-1α through the E3 ligase carboxy terminus of Hsp70-interacting protein and subsequent proteasomal degradation [15].

Hyperglycaemia not only inhibits HIF-1α stability, but also represses the transactivation activity of HIF-1 (Fig. 1c). Hyperglycaemia inhibits the activity of both N-terminal and C-terminal transactivation domains of HIF-1α [11]. This is at least partially mediated by methylglyoxal modification of p300, which inhibits its recruitment to HIF-1 [17]. Methylglyoxal modification of HIF-1α can also inhibit the hetero-dimerisation of HIF-1, thus repressing HIF-1 function [18].

Although HIF-1 inhibition by hyperglycaemia has been shown in several studies, paradoxically, the activation of HIF-1 signalling by high glucose through a carbohydrate response element binding protein-mediated mechanism has been reported in glomerular mesangial cells, indicating a cell context-specific regulation of HIF-1 in diabetes [19].

Besides hyperglycaemia, hyperlipidaemia is a common feature of type 2 diabetes. During hypoxia in cardiomyocytes, high levels of fatty acids (palmitate or oleate) have been shown to inhibit succinate generation from glycolysis, which, in turn, represses HIF-1 activation by facilitating the PHD-dependent degradation of HIF-1α [14]. Whether this mechanism is specific for cardiomyocytes needs to be determined, since in most cells glycolysis is activated in diabetes. Metabolites from the citric acid cycle have been shown to regulate PHD and other 2-oxoglutarate-dependent enzymes; their potential role in mediating the dysregulation of HIF in diabetes warrants further investigation.

## Hypoxia and HIFs in diabetes and diabetes complications

Accumulating evidence suggests that hypoxia and inappropriate responses to hypoxia due to dysregulated HIF-1 signalling are important pathogenic factors, occurring both in tissues central for the development of diabetes (pancreatic beta cells and adipose tissue) and in tissues susceptible to diabetes complications (nerves, retina, heart, blood vessels, kidney and wounds).

### HIFs and impaired wound healing in diabetes

The pathogenic relevance of HIF-1 inhibition in diabetes was initially observed in diabetic wounds [11, 12]. Inhibited HIF-1 signalling contributes to impaired wound healing in diabetes, with induction of HIF-1 function promoting wound healing by increasing angiogenesis and fibroblast proliferation and migration in mouse models of diabetes [11, 20, 21]. As an iron-chelating agent clinically used to treat iron toxicity, deferoxamine (desferrioxamine) can reduce oxidative stress and induce HIF-1 activation, thereby accelerating diabetic wound healing [20]. A topical drug delivery system has been recently optimised and a clinical trial (ClinicalTrials.gov registration no. NCT03137966) is planned to test its efficacy in patients with diabetic foot ulcers.

### HIFs and diabetic nephropathy

Hypoxia is present in the kidney of individuals with type 1 diabetes and type 2 diabetes [22, 23]; in animal models it is found as early as three days after the induction of diabetes, predominantly in the medullary region (reviewed recently in [8]). Hypoxia in renal tubules is the driving force for tubular atrophy and interstitial fibrosis, which can further reinforce glomerular pathology during the development of diabetic nephropathy [24]. Renal tubular hypoxia is mainly attributable to increased oxygen consumption due to increased flux through the Na^+^–glucose cotransporter and increased mitochondrial uncoupling-induced leak respiration [8]. Tubular hypoxia promotes extracellular matrix expansion, resulting in further decreases in oxygen delivery and the initiation of a vicious cycle that contributes to the development of diabetic nephropathy [24].

The regulation of HIF-1 in diabetic kidney depends on the cell type. In mesangial cells, high glucose increases the expression of HIF-1α and its target gene *ADAM17*, which accelerates renal fibrosis [19, 25]. However, HIF-1 is inhibited by high glucose levels in proximal tubular cells in hypoxia, which can be reversed by PHD inhibition and VHL deficiency [16, 26]. Overall, HIF-1 activation in the diabetic kidney is submaximal relative to the degree of hypoxia. Although absolute HIF-1α levels in diabetic kidney may remain unchanged or even increase, they are significantly lower relative to those in profound hypoxia [7]. Indeed, HIF-1 induction by PHD inhibitors can prevent the progression of diabetic nephropathy in animal models of both type 1 and type 2 diabetes [8, 27].

### HIFs and diabetic hearts

Cardiovascular disorders, including CHD, heart failure and diabetic cardiomyopathy, are the leading cause of mortality in individuals with diabetes and their prognosis is poor after myocardial infarction or heart failure.

Properly activated HIF-1 signalling is vital for cardiac survival after myocardial ischaemia or heart failure [28]. However, HIF-1 signalling is inhibited in poorly controlled diabetes in direct connection with metabolic control [13]. Recently, fatty acids were suggested to play an additional role in HIF repression in cardiomyocytes with a type 2 diabetes-like phenotype [14]. Reversal of HIF-1 inhibition in diabetic hearts by pharmacological inhibition of PHD improved cardiac recovery after ischaemia in a rat model of type 2 diabetes [14].

Diabetes accelerates atherosclerosis by inducing endothelial dysfunction, inter-plaque haemorrhage and plague destabilisation. Although HIF-1 has been reported to have both beneficial and pathological roles in experimental atherosclerosis, genetic and pharmacological inhibition of PHD protects against the development of atherosclerosis in high-fat-diet-fed LDL receptor-deficient mice [29].

### HIFs and diabetic retinopathy

Diabetic retinopathy has a progressive evolution. The initial, non-proliferative, phase is characterised by a loss of pericyte function resulting in microvascular abnormalities. These lead to hypoxia, an increased expression of angiogenic factors and subsequent neovascularisation, which characterise what is known as the proliferative phase. While the detrimental role of HIF-1 as a central stimulator of angiogenesis in the proliferative phase of diabetic retinopathy is established, proper HIF-1 function during the early stage of diabetic retinopathy is protective, due to its anti-inflammatory, antiapoptotic and antioxidative effects [30]. Indeed, the *HIF-1A* (also known as *HIF1A*) Pro582Ser polymorphism, which is resistant to inhibition by hyperglycaemia, is protective against the development of severe diabetic retinopathy [31].

### HIFs in adipose tissue and obesity

Hypoxia in adipose tissue is an early event in the course of obesity and leads to dysregulated adipokine production, inflammation and the metabolic syndrome. However, the role of HIF signalling in the development of obesity and metabolic disease is still controversial.

Some studies suggest a detrimental role of HIF activation in the pathogenesis of obesity and metabolic diseases. Exposure to hypoxia inhibits insulin signalling in adipocytes through HIF-1 and HIF-2 [32]. Genetic or pharmacological HIF-1 inactivation can prevent or reverse obesity-induced inflammation and insulin resistance [10, 33]. This is confirmed by data showing insulin resistance and adipose tissue fibrosis in transgenic mice overexpressing *Hif1a* [34].

However, recent studies using genetic or pharmacological PHD inhibition revealed a beneficial role of HIF activation in metabolic diseases. PHD2-hypomorphic mice, whether fed normal chow or a high-fat diet, displayed reduced adiposity, adipose tissue inflammation and hepatic steatosis, along with improved glucose tolerance and insulin sensitivity [35]. Adipose PHD2 deficiency or pharmacological PHD2 inhibition also increased adipose mass; however, reduced adipocyte lipolysis and normal glucose tolerance were also observed [36]. Moreover, the PHD inhibitor FG-4497 can reverse the metabolic dysfunction in aged or HDF-fed mice and in *ob*/*ob* mice [35].

The discrepancies between the results of these studies may stem from the distinct animal models used. However, they may also reflect the complex role of HIF signalling in metabolic diseases and stress the fundamental importance of adequate HIF function (neither too much nor too little) for the maintenance of homeostasis in hypoxic adipose tissue. Further investigations are warranted to elucidate the role of HIF-1 in obesity and metabolic diseases.

### HIFs and pancreatic beta cell function

Pancreatic islets from diabetic mice are hypoxic and high glucose induces hypoxia in beta cells and in isolated islets [9].

The protective role of HIF signalling in islet function and survival is the subject of a recent, excellent review [37]. Several studies have reported a protective role of HIF signalling for islet function and survival [38, 39]. Knockdown of HIF-1α or HIF-1β in beta cells was shown to inhibit glucose-stimulated insulin release. Interestingly, reduced HIF-1α and HIF-1β expression have been observed in the islets of individuals with type 2 diabetes, suggesting that islet HIF-1 inhibition may be a pathogenic mechanism in type 2 diabetes [38, 40]. Indeed, mice with a beta cell-specific HIF-1α deletion are more susceptible to type 1 diabetes after exposure to coxsackieviruses or beta cell toxin [41]. HIF-1 is also activated in beta cells during the pre-diabetes period of type 1 diabetes, where it is suggested to have a protective role [42].

Conversely, other studies have pointed to a potentially deleterious effect of HIF-1 on islet function. For instance, VHL gene deletion induces beta cell dysfunction that can be reversed by deletion of HIF-1α. Moreover, mice with beta cell or pancreas-specific VHL knockout develop glucose intolerance with impaired insulin secretion [43–45]. These results are in line with a recent study showing that mice with beta cell-specific HIF-1β knockout are protected from high-fat-diet-induced diabetes [46], suggesting a detrimental role of HIF-1 activation in beta cell function.

Taken together, these results indicate an important role for HIF-1 in regulating beta cell function and glucose tolerance. They also point to the importance of balanced HIF-1 signalling for proper beta cell function. Extremely low or extremely high HIF-1 levels after HIF-1 deletion, homozygous VHL deletion, severe hypoxia or HIF-1α overexpression are deleterious, while an increase in HIF-1α in response to hypoxia is beneficial for beta cell function and glucose tolerance.

### *HIF-1A* polymorphism and diabetes

*The HIF-1A* Pro582Ser polymorphism confers resistance to hyperglycaemia-mediated inhibition of HIF-1 activity and protects against the development of diabetic nephropathy, severe diabetic retinopathy and diabetic foot ulcers [7, 31, 47]. The *HIF-1A* Pro582Ser polymorphism is also protective against the occurrence of diabetes in the Japanese population [48].

### HIFs and epigenetic regulation in diabetes

HIFs control the expression and/or activity of epigenetic regulators that facilitate adaptation to hypoxia. Epigenetic regulation, including DNA methylation, histone modification and non-coding RNA, is also involved in the regulation and function of HIF signalling [49] and the pathogenesis of diabetes and its complications [50]. Epigenetic regulation seems to be an important mechanism underlying HIF regulation and function in response to hypoxia in diabetes. Its significance as a potential therapeutic target warrants further investigation.

## HIFs as therapeutic targets for diabetes and diabetes complications

As discussed above, hypoxia and impaired adaptive responses to hypoxia secondary to insufficient HIF-1 activation in diabetic tissues are fundamental pathogenic factors for the development of diabetes and diabetes complications. Therefore, strategies designed to increase HIF-1 signalling could lead to promising therapies for the treatment of diabetes and its complications.

Pharmacological induction of HIF-1 promotes wound healing in experimental diabetes models [11, 20, 21]. Recent preclinical studies in diabetic animal models have shown that PHD inhibition can also prevent the progression of diabetic nephropathy [8, 27] and atherosclerosis [29], protect the ischaemic heart [14, 51] and peripheral neuron [52], and improve cognitive function [53]. Some studies also show that PHD inhibition is beneficial for the prevention and treatment of obesity and metabolic disorders [35, 36] and for improving beta cell function [37].

The prolyl hydroxylase inhibitor (HIF-PH inhibitor) roxadustat (FG-4592) has recently been approved for the treatment of anaemia caused by chronic kidney disease [54] and several other HIF stabilisers are undergoing clinical trials. However, the clinical therapeutic effects of PHD inhibitors on diabetes and diabetes complications needs further investigation.

While topical application of HIF inducers for diabetic foot ulcer has only minimal systemic effects, further mechanistic and translational research is required to identify the right dose, temporal window and tissue-specific application for systemic use of HIF inducers to minimise potential side effects. More efforts to decipher the regulation of HIF-1 signalling in diabetes may provide novel and more specific therapeutic targets as well as efficient biomarkers for the identification of individuals who are most likely to benefit from HIF-targeting therapy.

## Conclusion and perspectives

**Fig. 2:**
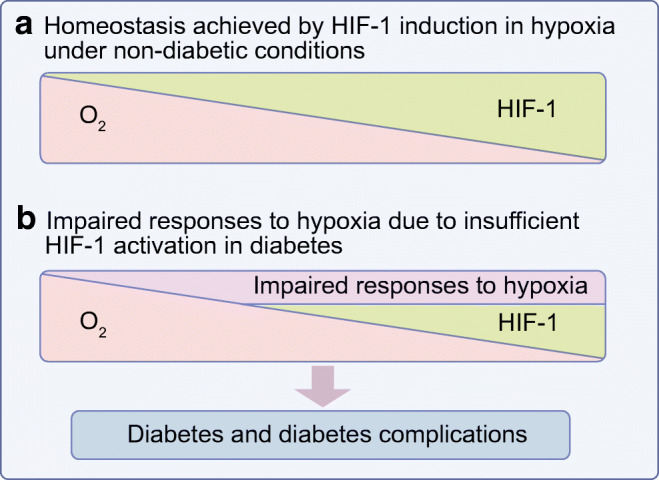
Impaired responses to hypoxia due to HIF-1 inhibition in diabetes contribute to the development of diabetes and diabetes complications. (**a**) Under non-diabetic conditions, HIF-1 signalling is induced in response to lowered oxygen levels, leading to homeostasis in hypoxia. (**b**) Under diabetic conditions, although tissues are more hypoxic, HIF-1 signalling is inhibited resulting in impaired adaptive responses to hypoxia, contributing to the development of diabetes and its complications. This figure is available as part of a downloadable slideset

Diabetic tissues are hypoxic; however, adaptive responses to hypoxia are impaired due to the dysregulation of HIF-1 signalling in diabetes. This contributes to the progression of diabetes and its complications, which can potentially be prevented or treated by modulating HIF-1 expression or activity (Fig. 2). Further mechanistic, translational and clinical research is warranted to identify specific and efficient hypoxia- or HIF-targeting therapies for diabetes and its complications. A summary of the main points of this review can be found in the text box ‘Take-home messages’.

This is an emerging field with contributions from many research groups. Due to space limits, we regret that it has not been possible to cite all relevant publications in this review.

## Supplementary Information

Slideset of figures (PPTX 214 kb)

## Abbreviations

HIF: Hypoxia-inducible factor
HRE: Hypoxia response element
Hsp: Heat shock protein
PHD: Prolyl hydroxylase domain protein
VHL: von Hippel–Lindau protein
