# Supplementary material for: Hypoxia and hypoxia-inducible factors in diabetes and its complications
Source: Diabetologia. 2021 Jan 26;64(4):709–16. doi: 10.1007/s00125-021-05380-z (PMC7940280; doi:10.1007/s00125-021-05380-z)
Supplement: Supplementary file 1 — (PPTX 214 kb) [file 125_2021_5380_MOESM1_ESM.pptx]

## Slide 1
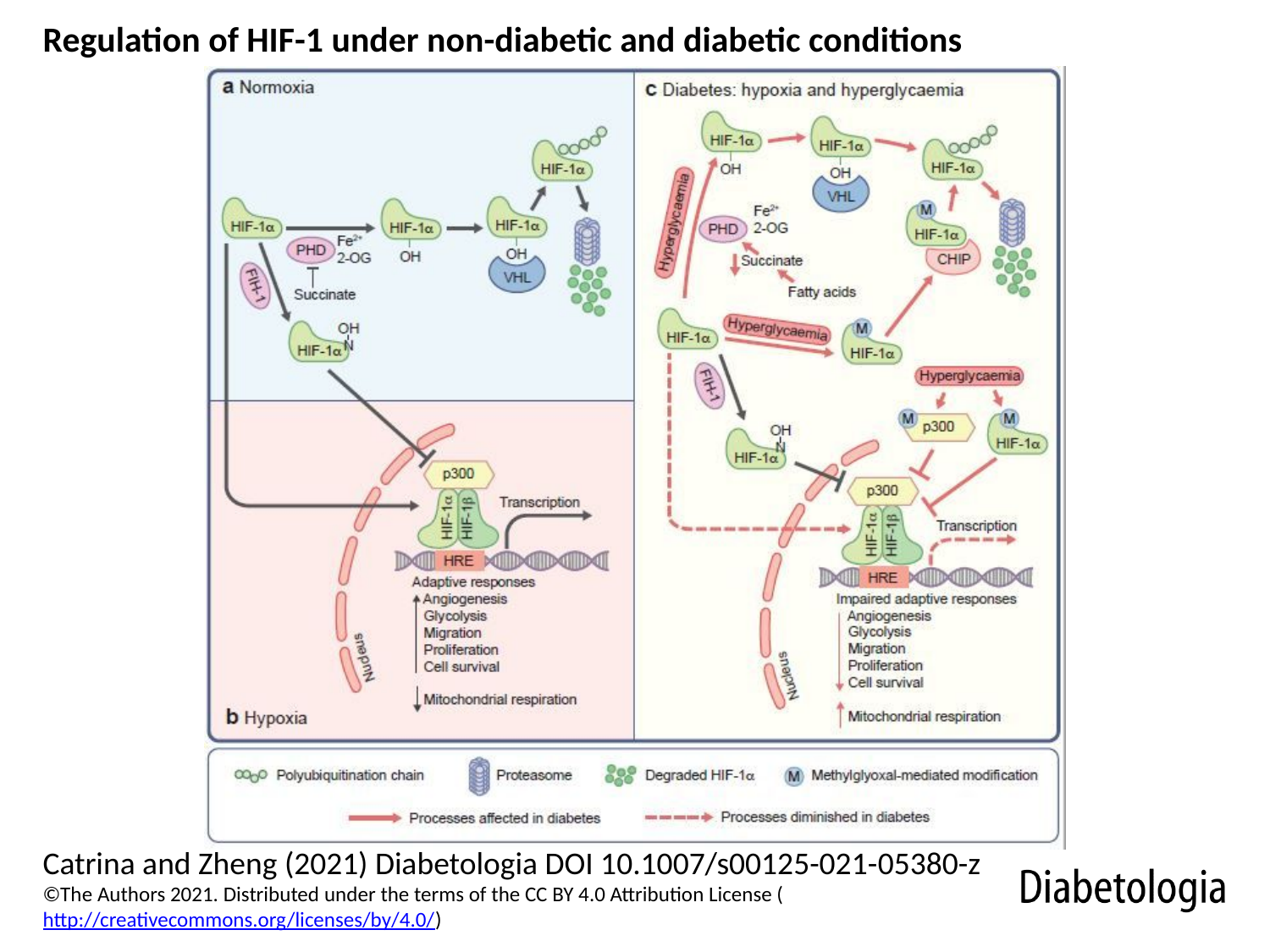

Regulation of HIF-1 under non-diabetic and diabetic conditions
Catrina and Zheng (2021) Diabetologia DOI 10.1007/s00125-021-05380-z
©The Authors 2021. Distributed under the terms of the CC BY 4.0 Attribution License (http://creativecommons.org/licenses/by/4.0/)

## Slide 2
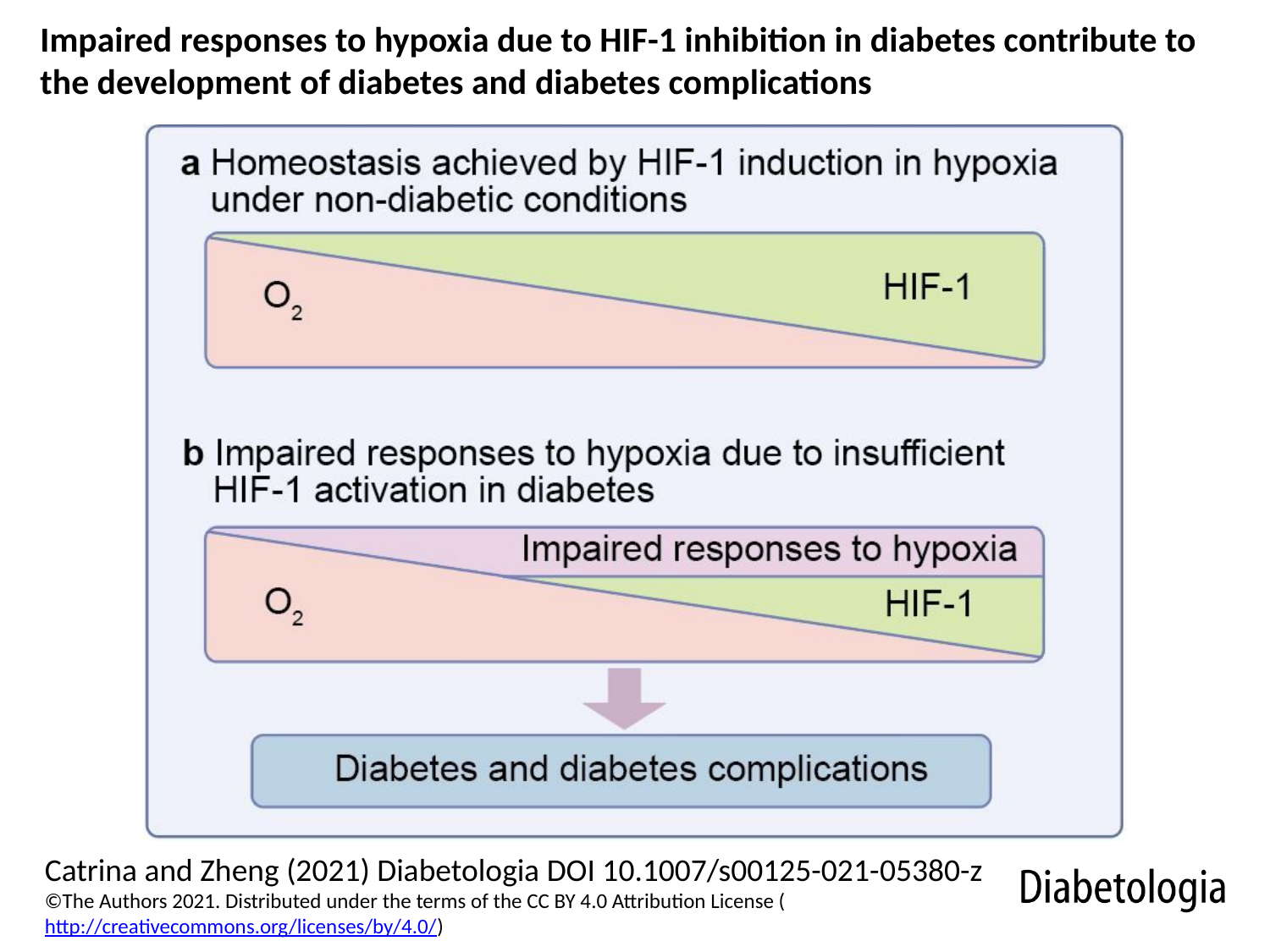

Impaired responses to hypoxia due to HIF-1 inhibition in diabetes contribute to the development of diabetes and diabetes complications
Catrina and Zheng (2021) Diabetologia DOI 10.1007/s00125-021-05380-z
©The Authors 2021. Distributed under the terms of the CC BY 4.0 Attribution License (http://creativecommons.org/licenses/by/4.0/)
